# Use of long-acting treatments in adult social care. Experience of an Acute Inpatient Mental Health Unit

**DOI:** 10.1192/j.eurpsy.2024.1195

**Published:** 2024-08-27

**Authors:** C. Rodríguez Gómez-Carreño, A. Sarrió Yuste

**Affiliations:** Psychiatry, HGUCR, Ciudad Real, Spain

## Abstract

**Introduction:**

In recents years, the number of social exclusion patients who go to the emergency room for psychiatric evaluation, has increased significantly. This fact may be due to the circumstances associated with migration: economic problems, house searching, moving away from the family origin,… These situations can cause stress before, during and after adaptation, which is a risk factor for presenting psychotic symptoms.

**Objectives:**

The objective of this study is present another alternative treatment for adults with social exclusion with psychotic symptoms or Psychotic Disorder. Presenting through a case of Acute Inpatient Mental Health Unit.

**Methods:**

A 25 year old men was referred to the emergency deparment due to an episode of agitation. As relevant psychiatric history, a previous admission to psychiatry’s hospitalitation with a diagnosis of Schizophrenia. Upon discharge, the patient has not been followed up in Mental Health, although he has gone to the emergency room on several ocassions where ir is reflected that no psychotic decompensation has been observed. He emigrated to Spain two years ago, since then he has been homeless, working intermittenly in agriculture.

At our assessment, after having ruled out consumption of toxic substances, the patient presented a neglected and cachetic appearance. He says that he is worried because some people can not see him and others can.

We admit the patient for study and treatment. Involuntary admission.

**Results:**

During the hospitalitation, a join approach was carried out with Social Work and it was decided to start depot treatment in order to promote therapeutic adherence. In this case, it was decided to apply paliperidone depot every sin month. For this, an induction regimen was followed: fisrt, monthly paliperdione 100mg depot was administered, 4 days later, monthly paliperidone 150mg depot and 4 days later, the biannual injection.

Other depot treatment alternatives would have been aripripazole or risperidone. However, the duration of the depot treatment is shorter than in the case of paliperidone, since today the presentation formulas are monthly and quarterly, respectively.

**Conclusions:**

Long-acting antipsychotics are an effective alternative for the treatment of patients with Schizophrenia, especially for those in whom we can not ensure good therapeutic adherence. In addition, the induction regimen allows treatment to be administered more quickly than that carried out in Mental Health outpatient programs, thus reducing the average hospital stay.

In recent years, great advances have been made in the treatment of psychotic symptoms thanks to depot drugs, which allows for numerous effective alternatives for the treatment of these patients. The figure of the Social Worker for the evaluation of the patient and subsequent follow-up is essential in this case.

**Disclosure of Interest:**

None Declared

